# Primary care teams’ experiences of delivering mental health care during the COVID-19 pandemic: a qualitative study

**DOI:** 10.1186/s12875-021-01496-8

**Published:** 2021-07-01

**Authors:** Rachelle Ashcroft, Catherine Donnelly, Maya Dancey, Sandeep Gill, Simon Lam, Toula Kourgiantakis, Keith Adamson, David Verrilli, Lisa Dolovich, Anne Kirvan, Kavita Mehta, Deepy Sur, Judith Belle Brown

**Affiliations:** 1 Factor-Inwentash Faculty of Social Work, University of Toronto, 246 Bloor Street West, Toronto, ON M5S 1V4 Canada; 2 Faculty of Health Sciences, Queen’s University, Kingston, ON Canada; 3 Telfer School of Management, University of Ottawa, Ottawa, ON Canada; 4 Association of Family Health Teams of Ontario, Toronto, ON Canada; 5 Village Family Health Team, Toronto, ON Canada; 6 Leslie Dan Faculty of Pharmacy, University of Toronto, Toronto, ON Canada; 7 Ontario Association of Social Workers, Toronto, ON Canada; 8 Department of Family Medicine, Schulich School of Medicine and Dentistry, Western University, London, ON Canada

**Keywords:** Primary care, Mental health care, COVID-19, Pandemic, Qualitative

## Abstract

**Background:**

Integrated primary care teams are ideally positioned to support the mental health care needs arising during the COVID-19 pandemic. Understanding how COVID-19 has affected mental health care delivery within primary care settings will be critical to inform future policy and practice decisions during the later phases of the pandemic and beyond. The objective of our study was to describe the impact of the COVID-19 pandemic on primary care teams’ delivery of mental health care.

**Methods:**

A qualitative study using focus groups conducted with primary care teams in Ontario, Canada. Focus group data was analysed using thematic analysis.

**Results:**

We conducted 11 focus groups with 10 primary care teams and a total of 48 participants. With respect to the impact of the COVID-19 pandemic on mental health care in primary care teams, we identified three key themes: i) the high demand for mental health care, ii) the rapid transformation to virtual care, and iii) the impact on providers.

**Conclusions:**

From the outset of the COVID-19 pandemic, primary care quickly responded to the rising mental health care demands of their patients. Despite the numerous challenges they faced with the rapid transition to virtual care, primary care teams have persevered. It is essential that policy and decision-makers take note of the toll that these demands have placed on providers. There is an immediate need to enhance primary care’s capacity for mental health care for the duration of the pandemic and beyond.

**Supplementary Information:**

The online version contains supplementary material available at 10.1186/s12875-021-01496-8.

## Background

The COVID-19 pandemic has exacerbated gaps in healthcare services and exposed inequities within many communities [1]. Primary care is the first-point of access in the healthcare system, promotes health equity, and has the potential to address many of these gaps by providing timely access to coordinated and integrated mental health care [2]. During the pandemic, primary care has maintained a foundational role in supporting the health and wellbeing of communities around the world [3, 4]. Despite the public health measures associated with the COVID-19 pandemic, such as lockdowns and quarantine procedures, primary care providers have continued to provide care for their patients including physical and mental health care needs [3, 4].

### Mental health and substance use during the COVID-19 pandemic

The COVID-19 pandemic has had extensive consequences on mental health and substance use worldwide. Prior to the COVID-19 pandemic, mental health conditions and substance use disorders were prevalent and leading causes of disability and illness related burden [5–7]. Mental health has worsened during the COVID-19 pandemic with increased stress, as well as higher rates of depression and anxiety [8–10]. In the United States, a study of over 300,000 individuals found that compared to 2019, Americans during the pandemic were three times more likely to have depressive or anxiety disorders [11]. Similarly, 40 to 50% of Canadians have experienced a deterioration of their mental health due to stress and anxiety associated with the pandemic [12, 13]. Early reports indicate that there are also elevated levels of substance use during the COVID-19 pandemic. In the United States, approximately one in 10 people reported that they started or increased their substance use during the COVID-19 pandemic [14]. Reports in the United States and Canada demonstrate higher rates of alcohol and cannabis use [14, 15]. In addition, there appears to be a heightened use of substances such as cocaine, fentanyl, and methamphetamine during the pandemic [16].

### Mental health care in primary care teams

Primary care is the first point of access for health services, and plays a central role in the coordination and integration of mental health services. Prior to the COVID-19 pandemic, primary care teams frequently provided care for common mental health conditions, such as depression and anxiety [17, 18]. Family physicians working in tandem with a range of health and mental health care providers in primary care teams have demonstrated increased capacity for patient care, and increased access to a broad range of health and mental health services for patients [19]. Team models of primary care facilitate access to mental health services including assessments, pharmacotherapy, psychotherapy, and care coordination needed for the recovery of common mental health conditions [2]. In addition, collaborative patient-centred primary care teams have improved patient outcomes [20, 21]. Early in the pandemic, primary care teams were instrumental for maintaining patients’ mental health [3]. Learning about primary care teams’ experiences delivering mental health care during the first two waves of the COVID-19 pandemic will help to prepare for the “echo pandemic” of mental health concerns [15].

### Study rationale

Our study sought to understand the impact of the COVID-19 pandemic on primary care teams’ delivery of mental health services in Ontario, Canada. Knowing what impact the COVID-19 pandemic has had on mental health care delivery within primary care settings will be critical to inform future policy and practice decisions during the later phases of the pandemic and beyond.

## Methods

### Design

We used a descriptive qualitative research design to guide sampling, data collection, and data analysis [22, 23]. Descriptive qualitative designs are valuable for health services research, particularly when ascertaining a description of phenomena is desirable [22, 23]. This aligned with our study which intended to describe what impact the COVID-19 pandemic had on mental health care delivery in primary care. Research team members involved in this study had different clinical, leadership, and disciplinary backgrounds representing social work, pharmacy, rehabilitation sciences, family medicine, and primary care health services research. The research team also included three knowledge users from two organizations – Association of Family Health Teams of Ontario and Ontario Association of Social Workers—that act as provincial leaders and advocates on behalf of primary care teams and mental health providers in Ontario. This research involving human participants was performed in accordance with the Declaration of Helsinki. We obtained ethics approval from the University of Toronto Research Ethics Board (REB Protocol #39,839).

### Setting

Family Health Teams (FHTs) are one model of team-based primary care in the province of Ontario, Canada’s most populous province [24]. A recognized type of “medical homes” [25], FHTs integrate physical, mental, and other behaviour health services through the inclusion of a range of different types of interprofessional providers on health care teams. There are 186 FHTs in Ontario, which is the largest team-based primary care model in Canada, providing services to approximately 25% of the province [26]. Although the composition of providers varies within each FHT, teams are typically comprised of family physicians, nurse practitioners, nurses, pharmacists, dietitians, as well as other types of providers [24]. Most FHTs include providers specifically for mental health care, including social workers (92% of FHTs), psychologists (25%), and general mental health workers (13%) [27]. The size of each FHT, types and number of services provided by family physicians and other interprofessional providers vary across FHTs [26].

### Sample and recruitment

We used a purposive sampling technique to engage a diversity of perspectives from various interprofessional health care providers (IHPs) that are involved in the provision of mental health services within FHTs. Potential participants were those working in FHTs who were able to participate in a virtual focus group and who offered mental health services. Our aim was to recruit FHTs from each of the five Ontario Health regions: West, Central, Toronto, East, and North [28]. We strived for representation from each of these five regions to i) include regional variation in terms of rural and urban; ii) reflect the varying diversity of populations in these regions; and iii) gain a provincial-wide understanding. We recruited FHTs by contacting the executive directors and inviting their team to participate. The first two teams from each of the five Ontario Health regions, who contacted the study’s research coordinator and identified an interest in participating in this study, were included in the study.

### Data collection

We developed a semi-structured interview guide (see supplementary file) and collected data using focus groups conducted via an online virtual video platform. We chose to use focus groups for data collection because focus groups are effective to generate information at the beginning of an inquiry [29], such as our inquiry on teams’ experiences during the pandemic. In addition, focus groups can enhance providers’ perspectives because of the dynamic nature of focus groups [29–31]. Finally we chose to use focus groups because they are an effective method to generate information from the perspective of diverse teams [29]. Focus groups were conducted with a combination of providers who worked together at the same FHT. Two research team members co-facilitated each of the focus groups. Focus groups were audio-recorded and transcribed verbatim immediately following the interview. Codes were assigned to protect the confidentiality of participants’ identities. All focus groups were conducted between October 2020 and December 2020.

### Data analysis

Data collection and data analysis occurred concurrently. Two researchers analyzed the data using thematic analysis [31]. The two researchers first became familiar with the data by reviewing the transcripts. Once familiar with the data, the two researchers generated initial codes in the data. When the initial coding was complete, the two researchers then met to confirm the coding structure with the data analysis sub-committee comprised of two other research team members. The data analysis sub-committee reviewed the codes for potential themes. Following which, the data analysis sub-committee named and defined the themes. During this process, we identified exemplar quotes to illustrate key themes. Then, all members of the research team had an opportunity to review the themes and provide input on the interpretation of the data at a virtual meeting. Finally, we related the themes and codes back to the study aims through the process of manuscript preparation. The coding process and development of themes was inductive. We established rigour and trustworthiness through reflexivity, prolonged engagement, and peer debriefing [32, 33]. We used NVivo12 to help organize the data and facilitate the analysis process.

## Results

We conducted 11 focus groups with 10 FHTs and a total of 48 participants. There was one focus group conducted at each of the nine FHTs, while we held two focus groups for one FHT to accommodate different team members’ conflicting schedules. The largest focus group included nine participants, although there was an overall average of four participants per focus group. There was geographic diversity as two FHTs represented each of the five Ontario Health regions. The participants (*N* = 48) represented a range of different types of healthcare providers and administrators (e.g. executive directors, program managers) (see Table 1).

**Table 1:** Participant role at FHT

**Participant Role**	**N**
Social Worker	20
Mental Health Therapist	10
Program Manager / Coordinator	9
Executive Director	4
Nursing (nurse practitioner, nurse health promoter, nursing manager)	3
Family Physician	2
Total	*N* = 48

With respect to the impact of the COVID-19 pandemic on mental health care in primary care teams, we identified three key themes: i) the high demand for mental health care, ii) the rapid transformation to virtual care, and iii) the impact on providers.

### High demand for mental health care

#### Worsening mental health

There was overwhelming agreement within all focus groups that patients’ mental health worsened during the COVID-19 pandemic. All focus groups discussed in depth the impact that the COVID-19 pandemic had on their patients:

*As time went on, people really declined…I have noticed a really big decline in mental health. I’m seeing more patients with mental health concerns, and those patients that were previously doing quite well are starting to decompensate*. (FG7, Nurse Practitioner)

All focus groups described seeing more patients struggling with anxiety during the pandemic: “*I’ve seen a lot more anxiety symptoms*” (FG4, Social Worker). Although not all patients were seeking care specifically for mental health, all focus groups noticed that the pandemic was broadly affecting people’s mental health. “*I think anecdotally it’s impacting a lot of people…I think it’s having an impact on them, and I see it indirectly*” (FG3, Family Physician). Focus groups discussed the need to adapt some patients’ treatment. For example, patients who previously, “*didn’t need medication before, now are maybe needing medication… [As well,] there are some newly presenting patients*” (FG7, Nurse Practitioner). In addition, some patients with past-histories of mental health difficulties were struggling: “*More people just need psychotherapy…because of the additional stress of COVID…. I’m getting a lot of people…they’re being referred back…just with all the extra stress of what’s going on right now…so then they’re coming back to see our psychiatrists*” (FG7, Psychiatry Intake Clerk).

#### Increased crises

All focus groups overwhelmingly agreed that they were seeing an increase in mental health crises among their patients. A social worker in one focus group noted that they were “*seeing…relatively stable clients suddenly go into crisis*” (FG10, Social worker). Most focus groups described an increase in suicidality during the COVID-19 pandemic, “*Last night in fact, I was on call, and I had an encounter with a patient and sent her to the hospital because she was suicidal*” (FG7, Family Physician). Another focus group discussed the impact that a recent suicide had on the team:

*We just had a suicide of a patient a couple weeks ago. His work was impacted by COVID…and then unfortunately he took his own life…His primary care provider had had him throughout his entire practice and came down to my office and was just in shock…. And then yesterday there was a call come through. Another person concerned about their spouse who was suicidal… So we’re seeing sort of this suicidal increase*. (FG9, Executive Director)

In addition, most focus groups – particularly those held in rural communities – noted an increase in crisis related to substance use amongst their patients. “*We’ve always had issues with addictions in our community, but now… it’s more obvious …the hospital is having more people being admitted for overdosing …We’re used to hearing about people overdosing, but now we’re actually knowing that this is happening*” (FG8, Program Manager). Similarly, another focus group in a rural community noted, “*We’ve seen a number of overdose deaths…we’ve certainly seen the number of overdoses increase*” (FG9, Social Worker).

#### Isolation, exhaustion, and fear

The conditions of the pandemic perpetuated isolation, exhaustion, and fear amongst patients. This sub-theme resonated with all focus groups. “*I’ve seen a lot more anxiety for sure, and a lot of isolation, and loneliness. And people…having to learn different coping skills because the coping skills they used to use, they’re not able to because of the restrictions*” (FG2, Social Worker). In another focus group, a family physician elaborated on how challenging it is for providers and patients not being able to rely on previous methods of coping or stress relief:

*We can fiddle with medications and such from the physician end of things, and we can have supportive chats…but that only goes so far... Patients who are more vulnerable, it is harder to come up with solutions that would help them. I would say there’s been more than a few times where I’m with a patient, and I’m struggling to figure out, ‘well, okay, what are we going to do here?’.... I’m feeling a little bit discouraged*. (FG6, Family Physician)

All focus groups described seeing patients exhausted, and noted that this became more evident to them as the pandemic persisted. “*The longer [the pandemic has] gone on, the more trying it’s been for people, and the more exhausted they feel, and the more they’ve…exhausted their strategies and their resources* (FG7, Social Worker). Further, another focus group expressed concerns with patients’ stress levels, “*Patients…are more stressed than ever!*” (FG6, Program Manager).

#### At-risk populations

Focus groups identified several types of patients that were at-risk of worsening mental health during the pandemic, including older adults, youth, and individuals living in rural communities. One participant stated, “*The people who are struggling more than others, are probably the people who are more vulnerable to being socially isolated, so seniors, the people with young children…and people with previous mental health [concerns], I would say are having a harder time than usual*” (FG7, Family Physician). Similarly, another focus group noted, “*I have several older clients…loneliness is becoming a major factor and some of them have taken a turn towards darker thoughts*” (FG11, Mental Health Therapist). A social worker noted, “*One thing I definitely noticed is a higher incidence of referrals for youth and senior population*” (FG1, Social Worker).

Many focus groups spoke about some of the mental health challenges that youth experienced during the pandemic. “*A ton of kids with anxiety. A lot of OCD, a lot of just generalized anxiety around the pandemic*” (FG3, Family Physician). Another participant noted a difference in their youth patients during the pandemic as compared to previous years: “*Teenagers I was working with…I think there was more…depression*” (FG2, Social Worker). Participants also observed an increase in crises among youth during the pandemic. “*Youth are suffering a little bit more from overdose and…depression*” (FG8, Social Worker).

Focus groups held in rural communities expressed concerns that individuals living in rural communities were particularly isolated during the pandemic, and thus, at increased risk for mental health difficulties. “*We’re isolated anyways here, and now this is kind of another layer of isolation*” (FG8, Social Worker). Additionally, “*because we’re rural…there’s been a lot more depression related to feeling really isolated*” (FG9, Executive Director). Some focus groups explained that rural and Northern patient populations experienced COVID-19 related stigma, leading to further isolation. For example, “*There seems to be…general stigma for COVID in a rural community than there would be in a more urban one…I’ve heard of some of the transactions in other rural communities where people had gotten COVID, and brought it into their community, and they’re pretty ridiculed*” (FG8, Social Worker).

#### Increased referrals and long wait-lists

All focus groups described the increased demand for mental health services since the onset of the COVID-19 pandemic. For example, “*mental health and social work are the two busiest programs right now and our referrals are coming in fast and furious*” (FG5, Program Coordinator). Increased referrals resonated with all focus groups: “*Our biggest intake for counseling was in August, it was almost tripled in August*” (FG9, Nurse Health Promoter). All focus groups agreed that the conditions of the COVID-19 pandemic led to increased demands for mental health services, “*We’ve been seeing more referrals – and not just from people who have pre-existing anxiety or depression, but new referrals coming in that are people that are specifically having a hard time with COVID*” (FG7, Social Worker).

As demand for mental health services increased, most focus groups agreed that waitlists became problematic. “*The need is definitely increasing. We’ve gotten a lot of referrals in the last month, so – we haven’t had a waitlist up to this point, but going forward we’re definitely going to, just because of the demand*” (FG3, Social Worker). Participants explained that the waitlists for mental health services also grew because of decreased access to community mental health resources during the pandemic. “*It’s getting more difficult now to bridge people or to connect them with community resources…because [community resources] have cut back their services*” (FG3, Family Physician).

#### Rapid transformation to virtual care

All focus groups spoke at length about having rapidly implemented virtual care – telephone and video appointments – at the onset of the pandemic. “*Our team did a great job of pivoting to provide virtual mental health care pretty much over a weekend, and went to phone based calls sessions…for individual sessions*” (FG5, Mental Health Therapist). Another participant explained, “*It’s all being done by phone so even their…therapy sessions, it’s over phone*” (FG10, Program Coordinator). Across most focus groups, telephone appointments were the most frequently used modality for mental health appointments during the COVID-19 pandemic. “*I would say 90% want telephon*e” (FG8, Social Worker). One of the reasons why some patients prefer telephone appointments is that: “*It’s easier for them to find privacy on a phone call than then on a video call*” (FG10, Social Worker). On the other hand, some focus groups mentioned using video appointments on occasionally. For example, “*As a physician, I do have access to video calls…if I know it’s a specific mental health appointment…I’ll do a video visit so I can actually have a face-to-face conversation with them*” (FG3, Family Physician).

Many focus groups discussed some of the challenges they encountered with the initial transition to virtual care. For example, not all types of treatment were easily adaptable for virtual delivery. “*I used to do an in-person anxiety and depression group… and that’s harder to facilitate…online*” (FG7, Social Worker). One of the challenges raised in all focus groups was the lack of education and training in using virtual care modalities:

*It would be very useful…training people like myself... in how to perform virtual care, …using the hardware itself or the software itself…But also, training for how to perform counseling in a virtual setting, right? Right now, we are just trying to do what we normally do just over the phone, but maybe that’s better delivered, in a different way*. (FG9, Social Worker)

A few focus groups spoke about how they continued to see a small sub-set of patients for in-person care during the pandemic because virtual care was not effective for all patients. For example, “*People who have been…hard of hearing where virtual or phone was not really working that well…it’s harder on the phone…It has been better to meet where…we can see each other…We’re trying to be very selective about that though*” (FG11, Mental Health Therapist). Participants also noted that they used some in-person mental health appointments during the pandemic if they had concerns about a potential mental health crisis. A family physician explained, “*I’m on the phone, and I think somebody’s struggling with a mental health issue…there’s nothing stopping me from actually bringing them in for an in-person assessment*” (FG3, Family Physician).

#### Limited access to technology

One challenge that all focus groups spoke about was providers’ limited access to technology. For example, “*The lack of equipment at home…computer and things like that… was a challenge*” (FG10, Social Worker). In addition, all focus groups raised concerns that patients without access to technology may be disadvantaged. “*It’s challenging to get people information if they don’t have computers [to receive] information or resources*” (FG5, Mental Health Therapist). There was concordance across focus groups that virtual care may not be accessible to some disadvantaged populations, “*Vulnerable populations [may not] have access to technology, or isn’t comfortable over the phone*” (FG7, Social Worker). To address these challenges, one team was considering distributing tablets to patients: “*We’re thinking about…sending mobile data tablets to people’s houses for borrowing*” (FG11, Mental Health Therapist). 

#### Challenges in rural and remote communities

Rural and remote communities experienced challenges due to the lack of high-speed internet. One focus group explained, “*If [they] were able to get better access to high speed internet, that would make a tremendous difference*” (FG5, Mental Health Therapist). We even experienced connectivity problems when conducting one focus group, “*Like, this [virtual focus group], I disconnected once already and it’s like, I can barely see it because it’s very low-res, and it’s also jittery, so that’s one of the challenges of living in a Northern community…our internet service is pretty horrible here*” (FG8, Social Worker). Unreliable reception created significant challenges for telephone appointments: “*Phone reception and the lack of reliability…Cellphone reliability in a rural area, there’s so much of a session where I’m like, can you please say that again?…I didn’t get that part*” (FG11, Mental Health Therapist).

#### Impact on quality of care

Across all focus groups, virtual care improved access because it enabled some providers to be more available. “*We’re much more efficient at fitting people in…if I have a full day of phone calls…how difficult is it really for me to fit in an extra phone call? It’s not that difficult*” (FG3, Family Physician). Virtual care enhanced accessibility because patients did not need to travel for an appointment: “*People really appreciate the flexibility of not having to come into the office*” (FG10, Social Worker). Virtual care improved some patient’s ability to engage with care: “*I would say…moms who have young kids at home or people who…don’t want to drive…really enjoyed the option of phone calls*” (FG9, Nurse Health Promoter). All focus groups indicated that virtual care enhanced access for patients with anxiety: “*I have had people tell me that they wouldn’t have really access these services unless they were being offered virtually. Those people are people with high levels of anxiety who have anxiety about coming into the office*” (FG10, Social Worker). Some focus groups suggested that patients experienced less fear of stigma with virtual care, and that enhanced access for mental health services: “*I’ve had a couple of patients say too, that doing it over the phone, they find less stigmatizing…Patients say that they don’t have to worry about running into anyone*” (FG2, Social Worker). Additionally, many focus groups noted that virtual care helped enhance continuity of care. “*I’m discovering when I talk to them… they’re up at their cottage, or they’ve moved…or temporarily relocated…now I’m able to still remain connected with them through a telephone appointment. And so the continuity of care maybe is a little bit better*” (FG3, Nurse Practitioner).

Focus groups raised two concerns about the person-centeredness of virtual care. First, there were concerns that virtual care impeded the therapeutic relationship. “*There are challenges with that…connection is really lost*” (FG5, Mental Health Therapist). As well, there can be challenges with the nuances of communication during virtual psychotherapy, in particular. “*I do notice sometimes on the phone there’s a lot of pauses…like I thought they were going to say something, but then they were waiting for me. So, there’s been a lot of like disconnection*” (FG4, Social Worker). Additionally, focus groups raised concerns that the lack of visual cues limited their assessment ability. “*The biggest challenge is the transition and adjustment to going virtual and phone based and not having, especially with the phone…not having the same amount or type of information that I would get from in person*” (FG5, Mental Health Therapist).

### Impact on providers

#### Provider roles

Focus groups identified three ways that the pandemic affected provider roles: i) new professional responsibilities, ii) increased workload, and iii) the need to be innovative. Most focus groups described starting new patient care activities since the onset of the pandemic. First, most teams initiated check-in calls to patients. “*We were doing wellness checks with people at the beginning of the pandemic…We were calling and just following up on mental health and how they were doing*” (FG3, Social Worker). Some focus groups also conducted targeted wellness checks, “*There was a list of vulnerable patients that were put together, whether they be older adults, or lacked support in their own community, living on their own. And we would call to check in*” (FG1, Social Worker). For some focus groups, check-in calls were a strategy used when wait-lists for mental health services grew: “*They’ve had to change how they’re delivering… They are doing more check-ins…. It’s more just to say: how’s your mood? How you doing, just dealing with some of the psychosocial issues*” (FG4, Social Worker). These check-in calls were meaningful to patients. “*Talking with the patients…I think they really value the check-in calls…because it just shows that you know they’re not being forgotten about*” (FG11, Program Coordinator). New professional responsibilities also emerged because of the lack of in-person administrative support. “*Not having some of that clerical support has also been a big challenge because they would [help with] my waitlists…, even just booking appointments, I’m doing that all from home without…clerical support*” (FG1, Social Worker). This resonated with another focus group: “*The other challenge…was just not feeling as efficient in…my work because I don’t have the same access to [administrative] resources… just the added layer of work…so there’s a lot of extra I’m finding now*” (FG4, Mental Health Therapist).

All focus groups experienced an increased workload. Prior to the COVID-19 pandemic, the “no-show rate” for mental health appointments was high, so providers’ scheduled patients back-to-back. Since the pandemic, most participants indicated that “*I’m getting less no-shows, because people are able to just do it from the comfort of their home*” (FG3, Social Worker). Many focus groups explained that the challenge was that providers previously used the “no-show” time to complete in-direct patient care activities. “*It is much more intense in that you rarely get a no-show or a cancellation with telephone…So in terms of workload…now it’s back to back. Go, go, go, because nobody’s canceling*” (FG4, Social Worker).

All focus groups spoke about needing to be innovative to meet patients’ mental health needs during the pandemic. One focus group explained that they revised their triaging process for mental health services because of the high demand. “*We sort of became creative…That’s where [the Nurse Health Promoter] came in…and we came up with this sort of outline of how that would look…she does such a great job of being able to figure out what their needs are…rather than waiting to see the social worker*” (FG9, Executive Director). Another focus group explained how their team adapted their triaging approach and implemented a new psychoeducational group, for patients with COVID-19 related anxiety. “*We…try to within the two weeks deal with the case…versus wait-listing them for 6 months….Now we’re doing the group as sort of like a step one …so that’s how we sort of dealt with the influx of the referrals for COVID related anxiety*” (FG4, Social Worker). One team created asynchronous videos for patients that addressed various mental health topics:

*I recorded all the sessions…and I just refer people to these YouTube clips. They’ll go through the clips at their leisure, and then I’ll follow up with them for a post-test a week or two later…Just recently I recorded with our pharmacist a sleeplessness group that we’re just about to roll out.* (FG3, Social Worker)

#### Personal wellbeing

All focus groups overwhelmingly spoke about the personal toll they experienced and described feeling exhausted and isolated. One participant explained:

*I think the real palpable feel of COVID fatigue. People are tired, and I think the frontlines…have been helping patients all along, they’ve changed the way they work…I think when you initially do that…there’s a burst of energy…people feel that urgency, and they problem solve, and they come up with great solutions, but… there’s no end in sight… I think we’re seeing more mental health needs with staff as well…I have referred more to our EAP recently just because people are feeling just exhausted.* (FG7, Nursing Manager)

The theme of exhaustion resonated with another focus group, “*When it was March and there was all this like ‘rah rah health care workers!’…you kind of felt like you had this calling…and then the work continues on and…the team is feeling like very exhausted and run down…and you’re still like in the thick of it*” (FG9, Executive Director). Many focus groups expressed concerns of potential burnout: “*From a high level…I could see the clinicians…having more fatigue and burnout*” (FG4, Program Manager).

## Discussion

This study comprised an exploration of primary care teams’ delivery of mental health care during the COVID-19 pandemic. Our study identified three key themes: high demand for mental health care, rapid transformation to virtual care, and impact on provider.

### High demand for mental health care

Primary care teams are seeing the effects of the pandemic first-hand in their patients’ mental health [2]. Quantitatively, early reports showed a spike in rates of mental health conditions—including anxiety, depression, and suicidality – during the early waves of the COVID-19 pandemic [15, 34]. For example, one Canadian survey (*N* = 1803) showed that anxiety had almost quadrupled in adults while levels of depression have more than doubled since the onset of the pandemic [15]. Our study provides qualitative insights into the lived experiences of primary care teams grappling with the spike in mental health care demands during the COVID-19 pandemic. Participants in all of our focus groups described the increasing mental health challenges that their patients faced during the COVID-19 pandemic due in part to financial insecurity, job loss, and social isolation. Our findings are consistent with literature, highlighting the higher levels of social isolation and loneliness emerging from a period of social distancing and stay-at-home orders [35–37]. During this period of isolation and loneliness, primary care teams in our study maintained meaningful and intentional connections with their patients. Participants in our study spoke about how the interconnected relational nature of primary care acted as a lifeline for many patients struggling with mental health during the pandemic. Primary care teams maintained connected with their communities, which provided them with a unique understanding of the extent of the devastation caused by suicides, overdoses, and mental health crises during the COVID-19 pandemic. Furthermore, stigma is an important determinant of health and while our study did not explicitly study this topic, we recognize that addressing and reducing stigma occurs through education and raising awareness [38, 39]. The IHPs in this study identified areas that are often stigmatized such as mental illness, addictions, and suicide [40]. Primary care’s unique vantage point provides a rich understanding of the mental health care needs of their patients and communities, and a unique perspective not well captured elsewhere in the healthcare system.

Of utmost importance to the participants in our study was the accessibility of mental health care during the COVID-19 pandemic. The pandemic’s effect on individuals’ mental health highlights the importance of strengthening primary care teams’ capacity to address these rising demands that will continue long after the COVID-19 pandemic ends [41, 42]. In primary care teams, mental health care includes identification and diagnosis, assessments, pharmacotherapy, psychotherapy, structured follow-up, systems navigation, and case management [43]. Our study demonstrated that early in the pandemic, the demand focused on mental health skills that required knowledge aimed at assessment, outreach, symptom reduction, and crisis management. Although highly committed to caring for their patients’ mental health care needs, all primary care teams in our study struggled to keep up with the increasing demands arising during the pandemic. To meet the rising demand, primary care teams in our study quickly adopted creative and innovative approaches to reach patients and increase access to mental health care. For example, some primary care teams initiated virtual group programming on a range of mental health topics. In addition, other approaches included implementing new ways to triage patients in need of mental health services. Triaging is a challenging and complex task [44]. Development of guidelines for triaging of mental health services in primary care would help provide some consistency across teams. It will be important to continue to learn about the innovative ways that primary care teams are working with patients during this challenging period, and to consider how some of these approaches may continue to be useful following the pandemic.

Early in the pandemic, primary care teams in our study rapidly implemented wellness checks. Wellness checks were an entirely new activity whereby all primary care teams in our study reached out to their patients in the early waves of the pandemic, targeting their patients that they considered most vulnerable. Our study illustrates primary care teams’ ability for rapid implementation of coordinated health-promotion outreach activities [45]. Although the actual impact is currently unknown, these wellness checks were a significant source of support and likely mitigated crises for some patients. Although there is early data about some of these innovations beginning to emerge [3, 46], there is a need for more research to demonstrate primary care’s vast contributions to population health during the COVID-19 pandemic.

### Virtual care

With the onset of the pandemic, primary care teams quickly adapted by shifting their services online with little to no preparation [3, 8, 47].Virtual care refers to “any interaction between patients and/or members of their circle of care, occurring remotely, using any forms of communication or information technologies, with the aim of facilitating or maximizing the quality and effectiveness of patient care” ([48], p.4). Findings in our study demonstrated that telephone appointments were the most commonly used virtual care modality to provide mental health services during the pandemic. In our study, the use of video technology for mental health services was minimal aside from psychoeducational group programs. Prior to the COVID-19 pandemic, most primary care teams had never provided mental health services through virtual means. As such, there is a dire need for education and training of providers to ensure that they are well-equipped to delivery mental health care virtually.

Our participants identified some of the benefits and challenges associated with virtual care. Participants in our study found it difficult to conduct robust assessments and engage in therapeutic interactions without the richness of nonverbal cues that are inherent in face-to-face sessions. These findings are consistent with recent research investigating virtual mental health care during the COVID-19 pandemic [49]. Therapeutic relationships are foundational to person-centeredness in primary care [50] and the mental health recovery model [51]. Further research will help determine how virtual modalities can be used to build strong relationships and to provide high quality care that meets patients’ identified needs.

Findings in our study point to the need for continued consideration of the social and digital determinants of health in future planning regarding the delivery of mental health services within primary health care settings, both during the pandemic and afterwards. Study participants discussed how virtual care enhanced access to mental health services and promoted continuity of care, which is consistent with other studies [3, 46]. On the other hand, participants also discussed virtual health inequities and barriers to accessing virtual care, influenced by factors such as internet connectivity, cellphone reception, and the appropriateness of virtual care [52]. The evolution of virtual care needs to occur in a way that does not perpetuate widening disparities for some populations, including patients residing in rural and remote communities [46, 52]. The future of equitable virtual care in primary care is dependent on strong leadership with patients at the centre of decision-making [53].

### Providers

While we presented the three distinct themes identified in our study, they were inextricably linked with one another. Primary care teams in our study experienced high demands for mental health care—including heightened patient complexity and crises – without an increase of resources to meet these demands. It was evident during the focus group discussions, that patient suicides have had a profound impact on primary care teams. In addition, primary care teams’ workload increased with the rapid implementation of virtual care. Our study demonstrates that this has taken a toll on the mental health and wellbeing of primary care providers themselves. There are early reports that the pandemic conditions contributed to emotional distress; increased levels of stress, anxiety, and depression; and higher prevalence of insomnia for healthcare providers [54–58]. It was evident during our focus groups that primary care teams also experienced concerning levels of stress during the COVID-19 pandemic. Our study demonstrates some of the personal impact associated with doing mental health care work during the COVID-19 pandemic. Consistent with recent literature, providers in our study described challenges they faced working in a context of high demand for care and seeing more patients during the pandemic struggling with mental health, and in crisis [3]. Bohman et al. [59] describe three components of wellbeing for health care providers. First, efficiency of practice refers to the ability of providers to meet their workload demands. All focus groups emphasized, however, the need for greater investment in mental health care within primary care settings in order to meet the increasing demand. Second, personal resilience refers to the ability to maintain one’s self-care and uphold boundaries between personal and professional life. Third, fostering a culture of wellness refers to creating organizational contexts that are nurturing and promote connection, also needed to counteract the profound isolation experienced during the COVID-19 [60]. Attending to the wellbeing and recovery of primary care providers is essential during and beyond the COVID-19 pandemic.

### Limitations

We conducted this study with primary care teams representing one model of primary care in Ontario. In addition, we conducted our study during the second-wave of the COVID-19 pandemic.. We realize that the discussion may over-accentuate the negative effects of the COVID-19 pandemic on primary care teams’ delivery of mental health care. The providers and teams included in our study were also having their own personal experiences of surviving the pandemic. A shared traumatic reality—such as a pandemic – can have negative consequences for mental health providers including emotional distress related to the event, and feeling less effective and capable in their professional role [61, 62]. We anticipate that primary care team experiences with mental health care delivery will continue to evolve. Our study provides insight during one time period of the pandemic. Lastly, this study does not include the patient perspective which is an essential perspective in understanding mental health care delivery during the COVID-19 pandemic.

## Conclusion

From the outset of the COVID-19 pandemic, primary care quickly responded to the rising mental health care demands of their patients. Despite the numerous challenges they faced with the rapid transition to virtual care, primary care teams have persevered. It is essential that policy and decision-makers take note of the toll that these demands have placed on providers. There is an immediate need to enhance primary care’s capacity for mental health care for the duration of the pandemic and beyond.

## Supplementary Information

**Additional file 1.** Focus Group – Interview Guide.

## Data Availability

The datasets used and/or analyzed during the current study are available from the corresponding author on reasonable request.
