# Supplementary material for: Primary care teams’ experiences of delivering mental health care during the COVID-19 pandemic: a qualitative study
Source: BMC Fam Pract. 2021 Jul 1;22:143. doi: 10.1186/s12875-021-01496-8 (PMC8248293; doi:10.1186/s12875-021-01496-8)
Supplement: Supplementary file 1 — Additional file 1. Focus Group – Interview Guide. [file 12875_2021_1496_MOESM1_ESM.docx]

**Supplementary File**

**Focus Group – Interview Guide**

- What differences have you seen in your patients’ mental health care needs since COVID-19?
  - How did patients’ MH care needs change at the beginning of the pandemic (March-April)?
  - What about throughout the summer?
  - Vulnerable populations?
  - Racialized patients?
  - Children/youth?
  - Remote communities?
- What key challenges have you experienced in providing mental health care to your patients since COVID-19?
  - What specifically?
  - Working as a team?
  - Has your role changed in any way?
  - Assessment
  - Any other new challenges?
- Are there any new mental health services or changes to your roles since COVID-19?
  - Have any new activities or your roles changed from prior to the COVID-19 pandemic to now?
  - New ways of caring for unattached patients?
  - New community collaborations?
  - Innovative approaches to mental health care?
- From your experiences since COVID-19, what specific mental health services do you think work best through virtual care?
  - For example: Diagnosing, medication management, assessment, counselling, groups, other?
  - With specific populations?
  - When doesn’t virtual care work for MH?
  - What supports and resources are needed if you are going to do virtual mental health care post-COVID-19?
- What recommendations do you have to improve your team’s capacity to address your patients’ mental health care needs for the duration of the pandemic?
